# Biallelic Variants in TULP1 Are Associated with Heterogeneous Phenotypes of Retinal Dystrophy

**DOI:** 10.3390/ijms24032709

**Published:** 2023-01-31

**Authors:** Jan-Philipp Bodenbender, Valerio Marino, Leon Bethge, Katarina Stingl, Tobias B. Haack, Saskia Biskup, Susanne Kohl, Laura Kühlewein, Daniele Dell’Orco, Nicole Weisschuh

**Affiliations:** 1 Department for Ophthalmology, University Eye Hospital, University of Tübingen, 72076 Tübingen, Germany; 2 Section of Biological Chemistry, Department of Neurosciences, Biomedicine and Movement Sciences, University of Verona, 37124 Verona, Italy; 3 Institute of Medical Genetics and Applied Genomics, University of Tübingen, 72076 Tübingen, Germany; 4 Centre for Rare Diseases, University of Tübingen, 72076 Tübingen, Germany; 5 Praxis für Humangenetik, 72076 Tübingen, Germany; 6 CeGaT GmbH, 72076 Tübingen, Germany; 7 Department for Ophthalmology, Institute for Ophthalmic Research, University of Tübingen, 72076 Tübingen, Germany

**Keywords:** *TULP1*, inherited retinal degeneration, retinitis pigmentosa, Leber congenital amaurosis, cone dystrophy, cone-rod dystrophy, minigene assay, Tubby domain, structural analysis, unfolded protein response

## Abstract

Biallelic pathogenic variants in *TULP1* are mostly associated with severe rod-driven inherited retinal degeneration. In this study, we analyzed clinical heterogeneity in 17 patients and characterized the underlying biallelic variants in *TULP1*. All patients underwent thorough ophthalmological examinations. Minigene assays and structural analyses were performed to assess the consequences of splice variants and missense variants. Three patients were diagnosed with Leber congenital amaurosis, nine with early onset retinitis pigmentosa, two with retinitis pigmentosa with an onset in adulthood, one with cone dystrophy, and two with cone-rod dystrophy. Seventeen different alleles were identified, namely eight missense variants, six nonsense variants, one in-frame deletion variant, and two splice site variants. For the latter two, minigene assays revealed aberrant transcripts containing frameshifts and premature termination codons. Structural analysis and molecular modeling suggested different degrees of structural destabilization for the missense variants. In conclusion, we report the largest cohort of patients with *TULP1*-associated IRD published to date. Most of the patients exhibited rod-driven disease, yet a fraction of the patients exhibited cone-driven disease. Our data support the hypothesis that *TULP1* variants do not fold properly and thus trigger unfolded protein response, resulting in photoreceptor death.

## 1. Introduction

Inherited retinal degeneration (IRD) is a clinically and genetically heterogeneous disease group that affects approximately 1 in 3000 individuals in North America and Europe [1,2,3,4]. The clinical picture of IRD is rather heterogeneous, ranging from pan-retinal stationary disorders (e.g., congenital stationary night blindness, cone dysfunction), to degenerative diseases, such as retinitis pigmentosa (RP), to focal degenerative diseases, such as macular dystrophy. More than 270 genes have been associated with IRD (RetNet, https://sph.uth.edu/retnet; accessed on 1 December 2022), most of which have only a low prevalence (below 5%). One example is the *TULP1* gene (MIM#602280). Biallelic variants in this gene have been associated with various retinal diseases, including non-syndromic RP [5,6], early-onset RP (eoRP) [7,8], Leber congenital amaurosis (LCA) [9,10], cone dystrophy (CD) [11], and cone-rod dystrophy (CRD) [12], among others. The frequency of IRD cases caused by biallelic variants in *TULP1* is below 1% in European and North American IRD cohorts [13,14,15]. In Arab cohorts, however, it reaches up to 14%, reflecting founder mutations and a high degree of consanguinity [16]. As of November 2022, the Human Gene Mutation Database (HGMD) [17] lists 106 variants in *TULP1*, most of which are missense variants, but nonsense and splice site variants, as well as small insertions and deletions, have also been described.

The *TULP1* gene encodes for Tubby-related protein 1 (TULP1), a cytoplasmic, membrane-associated protein specifically expressed in photoreceptor cells [18,19] and localized in the proximity of the plasma membrane, where it interacts with F-actin [20] and phosphorylated phospholipids at the level of the inner segment, connecting cilium, and an outer limiting membrane. Like other members of the Tubby protein family, the C-terminal domain of TULP1 binds to high selectivity specific phosphoinositides [21], predominantly phosphatidylinositol 4,5-bisphosphate (PIP_2_) [22], a phospholipid component of plasma membranes acting as a substrate for signaling proteins and important for endocytosis. TULP1 is, therefore, expected to play a role in inositol triphosphate (IP_3_)/diacylglycerol (DAG) signaling pathways, although its specific role remains to be clarified. Previous studies on mouse models demonstrated that TULP1 may be a component of the cellular machinery designated to protein/vesicle transport such as that of rhodopsin and other photoreceptor disc components [23,24] from the inner to the outer segment through the connecting cilium [25,26], together with other cytoskeleton proteins such as dynamin-1 [27], microtubule-associated protein (MAP)1A and MAP1B [28], kinesin family member 3A and RIBEYE [29]. Functional interaction between TULP1 and RIBEYE is essential to maintain synaptic ribbon integrity in mouse photoreceptors [30]. In addition, TULP1 was also found to act as an eat-me signal, together with Tubby protein, for retinal pigmented epithelium cells and macrophages [31] through the interaction with MerTK [32], and to potentially act as a DNA-binding transcription factor [22,33].

Currently, there are no approved treatments for IRD associated with *TULP1*. Gene replacement therapy by subretinal administration of adeno-associated virus-delivered *TULP1* to *TULP1* knockout mice on postnatal days 2–3 showed efficient expression of TULP1 protein in photoreceptors but provided only marginal and transient functional benefit [34].

This study aimed to describe the clinical heterogeneity of *TULP1*-associated IRD in a cohort of 17 patients diagnosed with IRD and harboring biallelic variants in the *TULP1* gene. In addition, we performed minigene assays to analyze two splice variants, and we used a computational structural approach to examine the effects of 40 different missense variants on the apparent relative stability and affinity of apo and Inositol 1,4,5-triphosphate (IP_3_)-bound human TULP1.

## 2. Results

Seventeen patients with biallelic variants in *TULP1* were included in this study. The mean age of the patients was 31.2 years (range 6–80 years).

### 2.1. Clinical findings

Of the seventeen patients, three were diagnosed with LCA, nine with eoRP, two with RP with an onset in adulthood, one with CD, and two with CRD (Table 1). Ophthalmological findings were highly symmetrical in both eyes. Of the 34 eyes in our study, 29% (10/34) had visual acuity worse than 1.3 logMAR, corresponding to blindness according to the WHO definition [35]. According to the definition of legal blindness under German law, which also takes perimetry findings into account, 35% (12/34) were considered blind [36].

In the following subsections, we will report on the different phenotypes and present clinical data for each patient including genetic findings, functional testing, and multimodal imaging.

#### 2.1.1. Leber Congenital Amaurosis (LCA)

**Figure 1:**
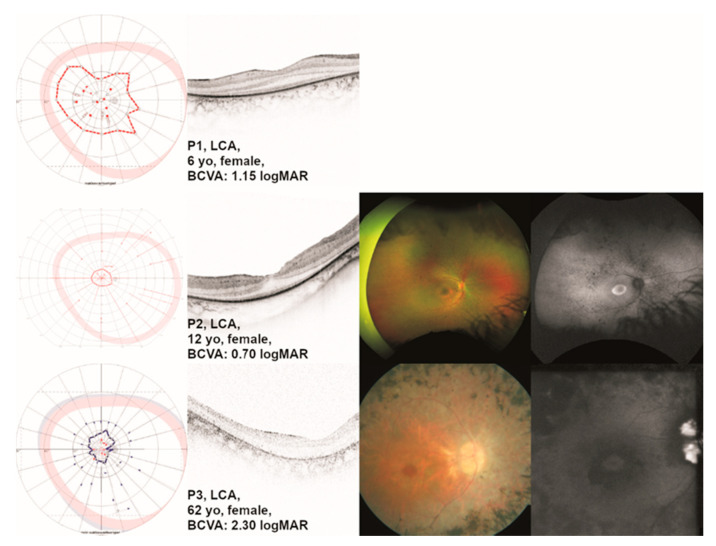
Clinical presentation of patients with *TULP1*-associated Leber congenital amaurosis (LCA). One eye per patient is listed by age at examination from top to bottom including (where available) the results of kinetic visual field (VF) testing, optical coherence tomography (OCT), fundus photography, and fundus autofluorescence (FAF) imaging. Gender and best-corrected visual acuity (BCVA) are also listed. Note the visible but altered ellipsoid zone (EZ line) on OCT in the two youngest patients (P1 and P2) compared to the older patient (P3), where the EZ line is no longer visible with outer retinal atrophy. Additionally, note the paracentral hyperautofluorescent ring on FAF in patient P2, whereas patient P3 shows paracentral hypoautofluorescence as in paracentral atrophy.

Patient P1 presented to us at the age of 6 years. She had been symptomatic from birth, exhibiting nystagmus and strabism. Patient P2 presented to us at the age of 12 years. She reported poor vision from birth on, accompanied by strabism and night blindness. Patient P3 presented to us at the age of 62 years. Her central vision had always been poor. She had night blindness and nystagmus from birth. Visual fields and ocular images are displayed in Figure 1.

#### 2.1.2. Early-Onset Retinitis Pigmentosa (eoRP)

**Figure 2:**
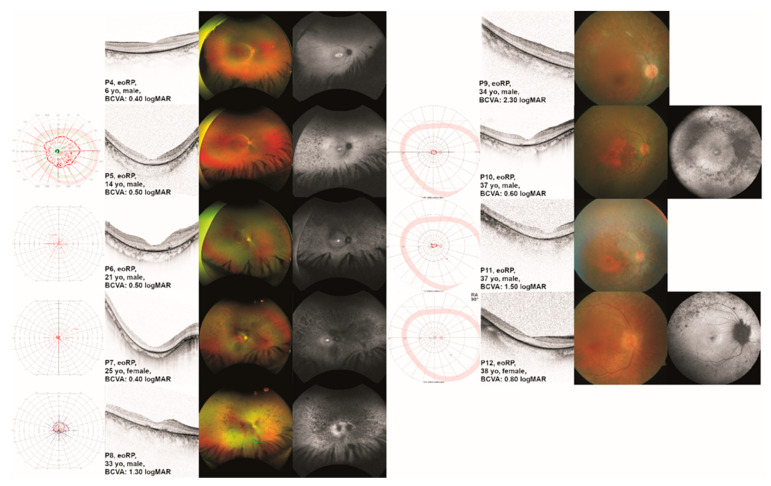
Clinical presentation of patients with *TULP1*-associated early-onset retinitis pigmentosa (eoRP). One eye per patient is listed by age at examination from top to bottom including (where available) the results of kinetic visual field (VF) testing, optical coherence tomography (OCT), fundus photography, and fundus autofluorescence (FAF) imaging. Gender and best-corrected visual acuity (BCVA) are also listed. Note the severely constricted VF in all patients except for P5, the second youngest patient. Additionally, note the visible but altered ellipsoid zone (EZ line) on OCT in the younger patients compared to the older patients. Note that only four of the patients show marked bone spicule pigmentation (P5, P6, P7, and P8), whereas the others show white atrophy. On FAF, the younger patients show central hyperautofluorescence. All patients but one (P4) showed hypoautofluorescence outside the arcades.

Patient P4 presented to us at the age of 6 years with night blindness, glare sensitivity, and difficulties in discriminating colors. His visual acuity had been poor since the age of 2 years. Patient P5 presented to us at the age of 14 years. He had received glasses at 2.5 years of age and reported increasing problems seeing at night. Visual field defects during the daytime were not reported at that time. Patient P6 presented to us at the age of 21 years. He had been diagnosed at the age of 5 years. His main early symptom was night blindness. His sister (P7) presented to us at the age of 25 years. She had been diagnosed at the age of 9 years. Her main early symptom was night blindness, as well. Patient P8 presented to us at the age of 33 years. He had been diagnosed with RP at the age of 13. He reported night blindness from 11 years on and reduced visual acuity. Patient P9 presented to us at the age of 34 years. He had been diagnosed with RP at the age of 6 years. He reported night blindness from early childhood on, as well as glare sensitivity, difficulties in discriminating colors, and reduced visual acuity. Patient P10 presented to us at the age of 37 years. He reported night blindness since childhood, and glare sensitivity and visual field defects since his early 30s. He reported difficulties in discriminating colors and decreasing central vision. His brother (P11) presented to us at the age of 37 years. He reported night blindness since childhood, and glare sensitivity and visual field defects since his early 30s. He reported difficulties in discriminating colors. Central vision had always been better in the left eye when compared to the right eye, and there was persistent nystagmus. Patient P12 presented to us at the age of 38 years. She had been diagnosed with RP before the age of 10 years. Her main early symptom was night blindness. Visual fields and ocular images are displayed in Figure 2.

#### 2.1.3. Retinitis Pigmentosa (RP)

**Figure 3:**
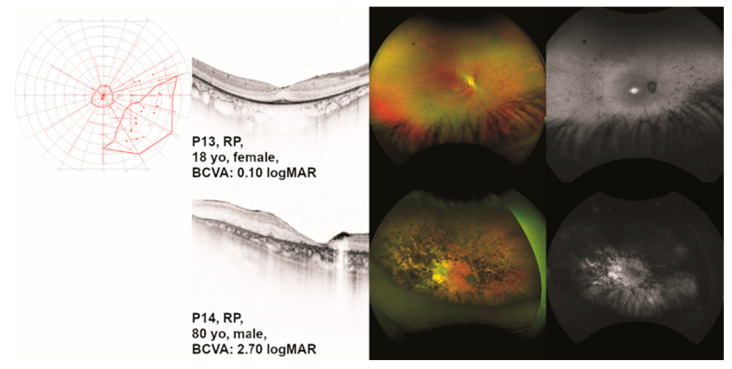
Clinical presentation of patients with *TULP1*-associated retinitis pigmentosa (RP). One eye per patient is listed by age at examination from top to bottom including (where available) the results of kinetic visual field (VF) testing, optical coherence tomography (OCT), fundus photography, and fundus autofluorescence (FAF) imaging. Gender and best-corrected visual acuity (BCVA) are also listed. Note the relatively conserved VF in patient P13. Additionally, note the RP-typical findings on OCT in the same patient when compared to the patients with a clinical diagnosis of LCA or eoRP (Figure 1, Figure 2). Note that only patient P14 shows marked bone spicule pigmentation. On FAF, patient P13 shows central hyperautofluorescence. In patient P14, even the central retina shows pathological autofluorescence as in far advanced disease.

Patient P13 presented to us at the age of 18 years. She reported night blindness. She had glasses since the age of 2 years and had temporarily occluded the right eye due to strabism. Patient P14 presented to us at the age of 80 years. He had been diagnosed with RP at the age of 44 years. Visual fields and ocular images are displayed in Figure 3.

#### 2.1.4. Cone Dystrophy (CD)

**Figure 4:**
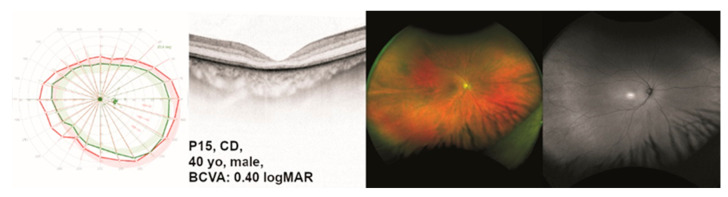
Clinical presentation of patient P15 with *TULP1*-associated cone dystrophy (CD). The results of kinetic visual field (VF) testing, optical coherence tomography (OCT), fundus photography, and fundus autofluorescence (FAF) imaging are shown from left to right. Gender and best-corrected visual acuity (BCVA) are also listed. Note the CD-typical findings in all shown modalities.

Patient P15 presented to us at the age of 40 years. Reduced visual acuity in both eyes had become apparent during a routine eye examination. He reported a BCVA of 20/25 and red color vision defects. At 40 years of age, he did not report glare sensitivity or night blindness. Visual fields and ocular images are displayed in Figure 4.

#### 2.1.5. Cone-Rod Dystrophy (CRD)

**Figure 5:**
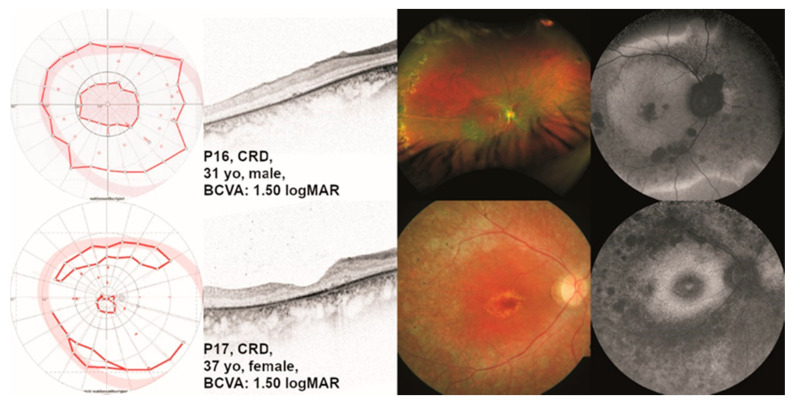
Clinical presentation of patients P16 and P17 with *TULP1*-associated cone-rod dystrophy (CRD). The results of kinetic visual field (VF) testing, optical coherence tomography (OCT), fundus photography, and fundus autofluorescence (FAF) imaging are shown from left to right. Gender and best-corrected visual acuity (BCVA) are also listed. Note the visual field in patient P16 with a large central scotoma. Additionally, note that the mid-peripheral and peripheral retina is relatively preserved on Optos and FAF imaging in this patient. Patient P17 shows retinal dystrophy with macular involvement.

Patient P16 presented to us at the age of 31 years. He had been diagnosed with retinal dystrophy at the age of 6 years. The main characteristics he reported were reading difficulties, color vision defects, glare sensitivity, and central visual field defects. At the age of 31 years, he did not report night blindness. Differential diagnoses included LCA and RP, but we concluded that CRD was the most appropriate diagnosis for this patient. Patient P17 presented to us at the age of 37 years. At the age of 14 years, she first noticed markedly decreased visual acuity. At 17 years of age, the patient had been diagnosed with macular dystrophy. She reported glare sensitivity and color blindness, no night blindness, but a “narrowed” visual field. Visual fields and ocular images are displayed in Figure 5.

### 2.2. Genetic Findings and Minigene Assays

Our patient cohort comprised 17 individuals from 15 families harboring putative disease-causing variants in *TULP1* (Table 1). Nine index patients were apparently homozygous for the respective variant. The six remaining index patients were proven compound heterozygous. Within our cohort, we identified seventeen different variant alleles, namely eight missense variants, six nonsense variants, one in-frame deletion variant, and two splice site variants (Table 2).

One of the splice site variants was localized at the invariable dinucleotide of the splice donor site of exon 14 (c.1495+1G>A). In general, variants at these positions do not require functional validation because the +1 and +2 positions are invariant in 99% of introns [38]. However, we decided to investigate the exact effect of the c.1495+1G>A variant to determine whether the induced mis-splicing disrupts the reading frame as this could have diagnostic implications. The second splice variant c.1496-6C>A we identified was the most frequent allele in our cohort (five alleles in four patients) and has been repeatedly observed in various patient cohorts [15,39,40,41,42]. The variant is located at the non-canonical acceptor site of exon 15; hence, it is classified as a variant of uncertain significance. To unambiguously classify this variant as non-pathogenic or pathogenic, we characterized it together with the c.1495+1G>A variant by minigene assays.

**Figure 6:**
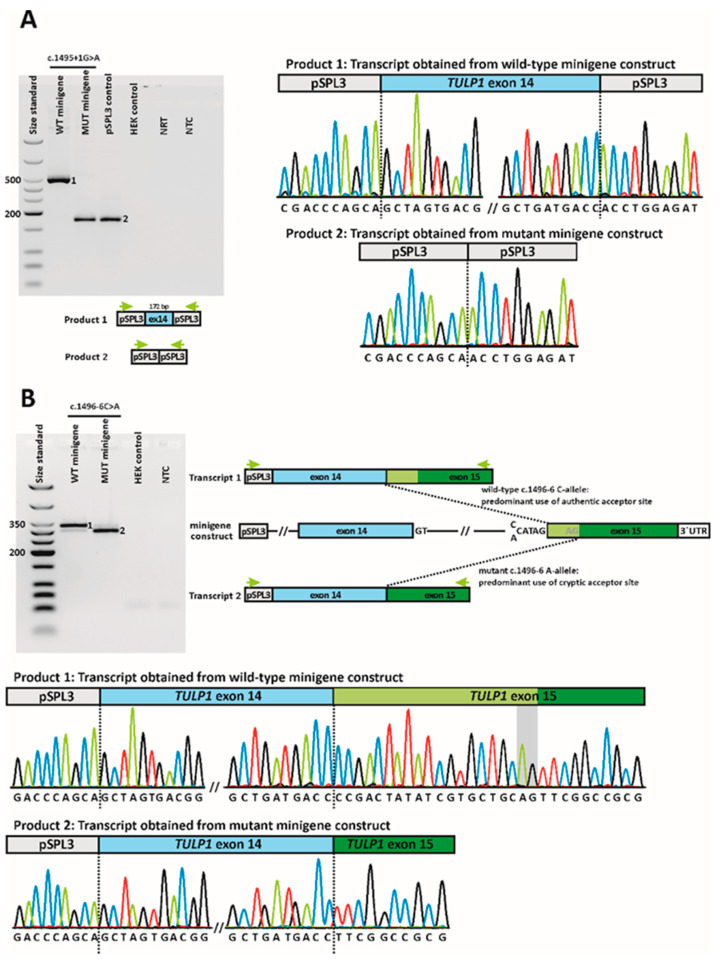
In vitro splice assays. (**A**) Variant c.1495+1G>A. Agarose gel electrophoresis of RT-PCR products is shown on the left. Leftmost lane, size standard (low molecular weight DNA ladder, NEB); lane 2, RT-PCR product of the wild-type allele; lane 3, RT-PCR product of the mutant allele; lane 4, transfection with empty pSPL3; lane 5, untransfected HEK293T cells; lane 6, no reverse transcriptase control (NRT); lane 7, no template control (NTC). Schemes of the amplified products are presented below the agarose gel. Gray boxes represent pSPL3 resident exons, and the blue box *TULP1* exon 14, respectively. Green arrows indicate the location of the RT-PCR primers. Sequence electropherograms are shown on the right side. Upper panel: the single RT-PCR product derived from transfection with the wild-type minigene construct corresponds to correct splicing (i.e., splicing of *TULP1* exon 14 between the pSPL3 resident exons). Lower panel: the smaller transcript expressed by cells transfected with the mutant minigene construct shows skipping of *TULP1* exon 14. (**B**) Variant c.1496-6C>A. The agarose gel shows the products from the second RT-PCR of the 3’RACE. Leftmost lane, size standard; lane 2, RT-PCR product of the wild-type allele; lane 3, RT-PCR product of the mutant allele; lane 4, untransfected HEK293T cells; lane 5, NTC. Schemes of the minigene constructs and the RT-PCR products are shown on the right. The first vector-resident exon is shown as a gray box. The blue and green boxes represent *TULP1* exon 14 and exon 15, respectively. The lighter shade in exon 15 represents its first 20 nucleotides with the cryptic acceptor. The 3´UTR is shown as a white box. Green arrows indicate the location of the RT-PCR primers. Sequence electropherograms after subcloning are shown below the agarose gel. Upper panel: the larger RT-PCR product obtained after transfection with the wild-type minigene construct shows that exon 14 is spliced to the authentic acceptor site of exon 15. Lower panel: the smaller RT-PCR product obtained after transfection with the mutant minigene construct shows that exon 14 is spliced to a cryptic acceptor site within exon 15, located 20 bp downstream from the authentic splice acceptor site. The AG dinucleotide that is part of the cryptic acceptor motif is highlighted in gray.

Variant c.1495+1G>A exerted a clear splicing defect in the minigene assays (Figure 6A). HEK293T cells transfected with the minigene construct harboring the mutant c.1495+1A-allele yielded a single RT-PCR product that was clearly smaller than the product from cells transfected with the wild-type c.1495+1G-allele. Moreover, the product size was the same as in cells transfected with the empty pSPL3 plasmid, indicating exon skipping (Figure 6A). Subsequent sequencing of the RT-PCR confirmed the skipping of exon 14 from the mutant transcript. The aberrant transcript would lead, if translated, to an insertion of 17 novel amino acids followed by a premature termination codon (PTC). Hence, the outcome on the amino acid sequence level can be described as p.(A442Pfs*18) (see Supplementary Figure S1A).

Furthermore, minigene assays revealed that the variant located at the non-canonical acceptor site of exon 15, c.1496-6C>A, activated a cryptic acceptor site, located 20 bp downstream from the authentic splice acceptor site of exon 15. On the agarose gel (Figure 6B), a major RT-PCR product was seen for the wild-type and the mutant allele, respectively, with a clearly discernible difference in size between the two RT-PCR products. With strong overexposure, an additional smaller RT-PCR product was visible in cells transfected with the wild-type allele. Conversely, a larger product was seen in the cells transfected with the mutant allele. Subcloning of RT-PCR products and subsequent sequencing showed that HEK293T cells transfected with the wild-type allele predominantly expressed the correctly spliced transcript, but also a small proportion of an additional transcript in which exon 14 was spliced to the cryptic acceptor site in exon 15 (Figure 6B). Conversely, cells transfected with the mutant allele predominantly expressed the aberrant transcript, but also expressed small amounts of the correctly spliced transcript (Figure 6B). The use of the cryptic acceptor site is predicted to lead to a frameshift and PTC (p.(P499Lfs*143), see Supplementary Figure S1B).

### 2.3. Molecular Modeling of Structural Variants

**Figure 7:**
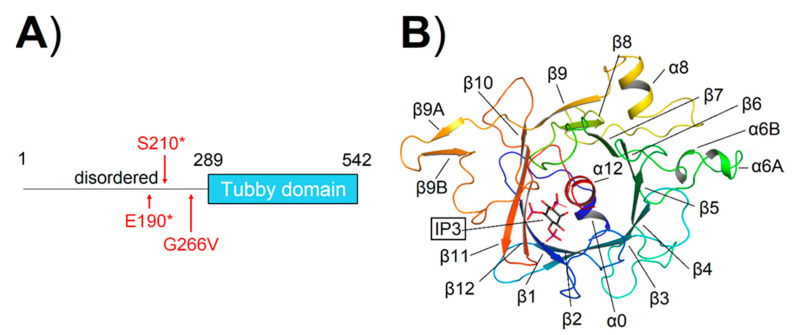
(**A**) Protein domain structure of TULP1; the position of the novel variants found in this study residing outside of the Tubby domain are indicated by red arrows. (**B**) Three-dimensional structure of IP_3_-bound TULP1. Protein structure is displayed as cartoons in a blue-to-red color scheme according to the sequence, secondary structure elements are labeled, and IP_3_ is framed and represented as sticks with C atoms in black, P atoms in violet, and O atoms in red.

*TULP1* encodes for a 60.6 kDa (542 residues) protein composed of a 289-residues, an N-terminal disordered region (Figure 7A), and a C-terminal Tubby domain [33] of 253 amino acids. The Tubby domain exhibits a peculiar fold, constituted by five α-helices (α0, α6A, α6B, α8, and α12) and 14 β-strands (β1 to β9, β9A, β9B, and β10 to β12) spatially arranged in an α-β barrel consisting of 12 β-strands (β1 to β12) surrounding the long, highly hydrophobic α-helix α12 (Figure 7B, Supplementary Video S1).

Out of the seventeen variants identified in our patient cohort, three are localized in the disordered region, namely E190*, S210*, and G266V, while the remaining fourteen variants map on the Tubby domain (Q301*, R342*, R342Q, N349K, R361*, P388Q, Q401*, R420S, R482Q, F491L, V503-G507del, R508H, c.1496-6C>A, and c.1495+1G>A), which represents a hotspot for *TULP1* variants.

As no structural information is currently available for the disordered region; we could not investigate the molecular effects of the G266V amino acid substitution. On the other hand, in the case of the two nonsense variants (E190* and S210*, Figure 7A), the entire Tubby domain would be missing. Thus, these protein products are most likely destined to degradation, leading to unfolded protein response (UPR) and, ultimately, endoplasmic reticulum (ER) stress, which was identified as the common pathological mechanism for the vast majority of *TULP1* mutations [43].

**Figure 8:**
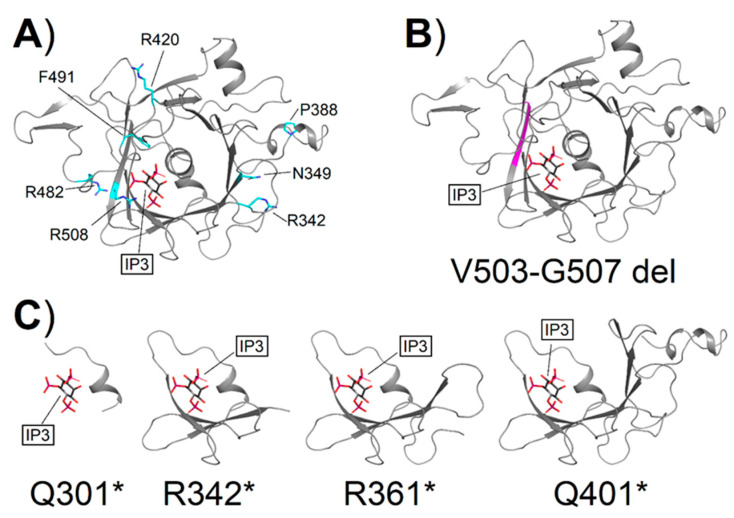
(**A**) Localization of TULP1 missense variants identified in this study. The three-dimensional structure of IP_3_-bound TULP1 is shown as gray cartoons. Residues whose mutations are associated with retinal degeneration in this study are labeled and represented as cyan sticks (C atoms) with O atoms in red and N atoms in blue. (**B**) The theoretical 3D structure of the IP_3_-bound TULP1 V503-G507 del variant, the region encompassing residues 503–507, is shown in magenta. (**C**) The theoretical 3D structure of IP_3_-bound TULP1 Q301* [44], R342*, R361* [45], and Q401* [15] nonsense variants. IP_3_ is framed and represented as sticks with C atoms in black, P atoms in violet, and O atoms in red.

To investigate the molecular consequences of the missense variants affecting the Tubby domain (Figure 8A, Supplementary Video S2), we performed an in silico analysis aimed at assessing the effects of the mutations on the apparent stability of the protein and the apparent affinity for IP_3_. All variants caused a destabilization of the folding of the protein, regardless of the presence of IP_3_ (Table 3), although with peculiarities. Indeed, variant F491L displayed the smallest variations in apparent stability in both the apo and the IP_3_-bound forms, and IP_3_ was found to slightly mitigate the variant-associated destabilization (5.44 ± 0.88 and 3.58 ± 1.05 kcal/mol for apo and IP_3_-bound, respectively, Table 3), similarly to the behavior exhibited by the R342Q substitution (6.97 ± 3.45 vs. 4.49 ± 1.53 kcal/mol, Table 3). Variant R508H was found to be the most detrimental to the apo form (29.32 ± 9.02 kcal/mol, Table 3), but in this case, no significant differences in ∆∆G_f_^app^ were found in the presence of IP_3_ (29.26 ± 8.29 kcal/mol, Table 3), probably due to the significantly lower affinity for IP_3_ of this variant (13.54 ± 3.30 kcal/mol, Table 3). Similar behavior was exhibited by variant R420S (15.58 ± 5.81 vs. 14.79 ± 3.46, Table 3), although the perturbation of IP_3_ affinity was less prominent (2.96 ± 2.60 kcal/mol, Table 3). Interestingly, the IP_3_-bound form of TULP1 was significantly more destabilized than its apo counterpart in the presence of variants P388Q (9.53 ± 0.30 vs. 17.97 ± 3.30 kcal/mol, Table 3), R482Q (24.32 ± 0.04 vs. 29.43 ± 1.70 kcal/mol, Table 3), and N349K (11.55 ± 0.04 vs. 40.10 ± 26.59 kcal/mol, Table 3), which proved to be the most destabilizing among the missense variants identified in our patient cohort. Specifically, despite the negative contribution to ∆∆G_f_^app^ provided by the solvation of the amine group of K349, such destabilization arises from the electrostatic repulsion of the positively charged K349 with the surrounding basic residues R342, K346, K369, and R371.

The computed apparent affinity for IP_3_ was less affected by the presence of disease-associated variants (Table 3), as shown by the significantly less consistent variations (∆∆G_b_^app^ <1 kcal/mol within errors) exhibited by the substitutions. The only exception to such behavior is represented by variant R508H, which resulted in a reduction in the apparent affinity of 13.54 ± 3.30 kcal/mol, most likely due to its direct involvement in the coordination of IP_3_. Taken together, these results suggest that none of the *TULP1* variants observed in this cohort, except for R508H, should decrease the affinity of the TULP1 protein for the photoreceptor plasma membrane, since anchoring occurs through binding of the Tubby domain (C-terminal) to PIP_2_, which is chemically analogous to IP_3_ in terms of the polar interactions involved. Folding defects are the likely pathogenetic mechanism of all tested variants.

Similar conclusions could be drawn when the analysis was extended to 38 other known disease-associated *TULP1* variants (Supplementary Table S1, Supplementary Video S3). Indeed, most of the variants displayed varying degrees of protein structural destabilization regardless of the presence of IP_3_. The only exceptions were substitutions K489R (∆∆G_f_^app^ = −16.79 ± 4.35 and −6.91 ± 9.92 kcal/mol for apo and IP_3_-bound forms, respectively, Supplementary Table S1), A496T (∆∆G_f_^app^ = −3.16 ± 1.79 and −1.28 ± 3.16 kcal/mol for apo and IP_3_-bound forms, respectively, Supplementary Table S1), and I530M (∆∆G_f_^app^ = −3.00 ± 0.76 and −0.04 ± 0.08 kcal/mol for apo and IP_3_-bound forms, respectively, Supplementary Table S1). However, while K489R displayed a significant reduction in apparent IP_3_-affinity (∆∆G_b_^app^ = 14.24 ± 0.41 kcal/mol, Supplementary Table S1), A496T and I530M substitutions exhibited negligible variations (∆∆G_b_^app^ = 2.44 ± 3.43 and −1.06 ± 0.58 kcal/mol, respectively, Supplementary Table S1), which might suggest a slight stabilization of the interaction between TULP1 and the PIP_2_-containing membranes. However, it should be noted that the predicted increase in affinity for IP_3_ of variants N349K, I459K, and Q492R is counterbalanced by a significant (> 11.5 kcal/mol) destabilization of the protein folding, regardless of the presence of the ligand. Therefore, our data suggest that the pathological mechanism underlying the onset of retinal degeneration is a TULP1 folding defect, a conclusion that is supported by experimental results [43].

In the case of the novel deletion V503-G507 del (Figure 8B), the translated protein would lack four of the central residues in β11, resulting in a shorter β-strand that may prevent the assembly of the other β-strands surrounding helix α12, thus potentially leading to unfolding. The computational structural analysis of nonsense TULP1 variants suggests that folding impairment is the most likely pathological mechanism, as the absence of several secondary structure elements would prevent the correct spatial organization of α-helices and β-strands required for the formation of the α-β barrel architecture that is typical of Tubby proteins. In detail, aside from the N-terminal disordered domain, in variant Q301*, only the sequence relative to helix α0 would be retained. In variant R342*, the β-strands β1 and β2 would also be preserved, with the addition of β3 in variant R361*, and β4 and β5 in the case of the truncation at the level of Q401* (Figure 8C).

## 3. Discussion

The 17 patients in our study cohort revealed various phenotypes and associated clinical diagnoses. In the literature, we found clinical details of 61 patients (from 29 families) with *TULP1*-associated IRD [5,7,9,10,11,39,40,49,50,51,52,53,54,55,56]. Of those, thirty-seven had been diagnosed with LCA, eleven with eoRP, six with RP, two were diagnosed with cone dystrophy, and five with “*TULP1*-RD”. All in all, we found the same spectrum of diagnoses in our cohort. However, it is worth noting that only 30–35% of the eyes in our study showed visual acuity and/or visual field equivalent to blindness according to the WHO definition and/or the definition of legal blindness according to German law, although *TULP1*-associated IRD is commonly considered a severe phenotype [51,57].

Two patients in our study deserve a more detailed description. Patient P15 showed typical findings of CD, e.g., bilateral progressive loss of central vision, central hyperautofluorescence on FAF imaging, and reduced photopic ff-ERG responses, whereas responses were normal under scotopic testing conditions. However, he did not report onset in childhood or teenage years but rather later, and he did not suffer from photophobia when examined at the age of 40 years. Additionally, while exhibiting a paracentral loss of the EZ line on OCT imaging, the EZ line in the center of the macula was relatively preserved. He was found to be compound heterozygous for a nonsense variant (c.1201C>T;p.(Q401*)) and a missense variant (c.797G>T;p.(G266V)). An isolated cone phenotype has been described previously by Roosing and colleagues, who reported a patient who was homozygous for a missense variant (c.1258C>A;p.(R420S)) and whose clinical characteristics were largely consistent with those of CD [11]. Of note, the same variant was found in a homozygous state in a second unrelated patient in the same report [11]. However, this second patient displayed typical characteristics of CRD, indicating that the same variant can lead to different involvement of cone and rod photoreceptors [11]. Interestingly, the patient in the study of Roosing and colleagues who was diagnosed with CRD showed a clinical picture similar in appearance to another patient in our study, namely P16. The latter had typical findings of CRD in visual acuity and kinetic visual field, which had a defined central scotoma with normal outside borders. While ff-ERG responses were only slightly reduced under scotopic testing conditions, they were markedly reduced under photopic testing conditions. Like the CRD patient described by Roosing and colleagues, he displayed pathology confined to the posterior pole most pronounced in the macula with a surrounding hyperautofluorescent border separating the lesion from the preserved retina in the mid-periphery and periphery. Patient P16 in our study was homozygous for the missense variant c.1163C>A;p.(P388Q), which showed similar characteristics to the variant c.1258C>A;p.(R420S) in structural analysis (Table 3).

Nearly all missense variants identified in our study can be considered disease-causing considering their impact on structural stability and affinity (Table 3). Indeed, Lobo and colleagues demonstrated that *TULP1* variants R420P, I459K, and F491L express misfolded proteins that accumulate within the endoplasmic reticulum, thus resulting in the activation of the unfolded protein response (UPR) complex [43]. We predicted a prominent destabilization of the protein folding for these variants (Supplementary Table S1), as well as for all the novel missense variants in our patient cohort (Table 3). Therefore, we suggest that protein misfolding is the pathogenetic mechanism involved.

However, no such data could be obtained for variant c.797G>T;p.(G266V) because of its location outside the Tubby domain. The glycine residue is evolutionarily conserved (Supplementary Figure S2) but has a relatively high MAF (0.0007141) compared with other disease-causing variants (Table 2). The variant has been described previously in an IRD patient but without detailed information on genotype or phenotype [58]. Hence, at this stage, we cannot be entirely sure that variant c.797G>T;p.(G266V) is disease-causing.

Considering the two splice variants we identified in our cohort, we could demonstrate that variant c.1495+1G>A leads to skipping of exon 14, resulting in a frameshift and PTC (p.(A442Pfs*18)) and likely degradation of the mutant transcript by nonsense-mediated mRNA decay. Our splice assay for the c.1496-6C>A variant indicated that it activated a cryptic acceptor site, located 20 bp downstream from the authentic splice acceptor site of exon 15. The use of the cryptic acceptor site is predicted to lead to a frameshift and PTC (p.(Pro499Leufs*143)). In consequence, the 44 amino acids encoded by exon 15 are lost and replaced by 142 novel amino acid residues. We hypothesize that the new C-terminal stretch does not lead to any specific folding, which increases the chance of the chimeric protein being degraded, and/or perturb its molecular interactions.

We would like to emphasize that a small proportion of transcripts were correctly spliced in our splice assay for variant c.1496-6C>A. Hence, this variant should be considered a hypomorphic allele that may be unmasked as being deleterious in the homozygous state or the heterozygous state with another pathogenic allele. In our cohort of patients, we observed the variant once in a homozygous state, while three patients were heterozygous and harbored an additional pathogenic allele (Table 1).

Of note, our splice assay for variant c.1496-6C>A also indicated that a small fraction of HEK293T cells transfected with the wild-type minigene construct expressed transcripts that used the cryptic exonic acceptor. While we could not find an annotated transcript lacking the first twenty nucleotides of exon 15 in any of the three common genome browsers NCBI, UCSC, and Ensembl, we saw evidence of this transcript in RNAseq data from the human retina [59]. Manual inspection of RNAseq data from three retinal samples with high coverage using the Integrative Genomics Viewer [60] revealed that a small fraction of reads (<1%) showed a junction between the donor site of exon 14 and the cryptic acceptor site in exon 15. This implies that the cryptic exonic acceptor is used in a minority of transcripts in vivo. Given the predicted consequences of the chimeric protein, it seems unlikely that the mutant transcript has a specific function. We hypothesize that it is merely a consequence of stochastic noise in the splicing machinery and has no functional significance [61]. According to ACMG guidelines [62], we classified the c.1496-6C>A variant as likely pathogenic, as per the following criteria: PS3, strong evidence based on minigene assays; PM2, moderate evidence based on the extremely low frequency of the variant in the large genome population database gnomAD (23/280,154); and PM3, moderate evidence based on the *trans* configuration with a pathogenic variant on the other allele.

## 4. Materials and Methods

### 4.1. Ophthalmological Testing

The study included 17 patients. All patients were examined at the Centre for Ophthalmology of the University of Tübingen, Germany, a tertiary referral center.

The ophthalmological examination included best-corrected visual acuity (BCVA), slit-lamp and dilated fundus examination, fundus photography, spectral domain optical coherence tomography (OCT), and fundus autofluorescence (FAF) imaging (Spectralis^®^ HRA+OCT, Heidelberg Engineering GmbH, Heidelberg, Germany), a semi-automated 90° kinetic visual field (VF) exam with targets lll4e and I4e (Octopus 900, Haag-Streit, Wedel, Germany), and full-field electroretinography (ff-ERG), according to ISCEV standards (Espion, Diagnosys, Lowell, MA, USA). BCVA was converted to logMAR visual acuity [63]. The most likely diagnosis was determined based on the patient’s first and late symptoms, findings from psychophysical and functional testing, and imaging.

### 4.2. Genetic Diagnostic Testing

Genetic diagnostic testing of patients in this study was performed using either a diagnostic gene panel for inherited eye diseases or by genome sequencing [15,64]. Genomic coordinates given in this manuscript are based on the GRCh38 genome (hg38). Variant nomenclature is in accordance with Human Genome Variation Society recommendations [65] and based on GenBank accession numbers NM_003322.6 and NP_003313.3, with nucleotide one being the first nucleotide of the translation initiation codon ATG.

### 4.3. In Vitro Splice Assays

In vitro splice assays were based on the pSPL3 exon trapping vector, as described previously [66]. Briefly, for the analysis of the c.1495+1G>A variant, a genomic segment of the *TULP1* gene (GrCh38/hg38 chr6:35,499,562–35,500,351; corresponding to exon 14 and flanking intronic sequences) was amplified from genomic DNA from a healthy control subject using a proofreading polymerase and cloned into the pSPL3 minigene plasmid vector. After verifying that this wild-type minigene construct generated normally spliced RNA, the c.1495+1G>A variant was introduced by site-directed mutagenesis [67]. Sanger sequencing was used to verify the presence of the introduced substitution and the integrity of the minigene. The resulting minigene constructs in their wild-type and mutant versions were used to transfect HEK293T/17 cells (ATCC^®^ CRL-11268™), which were then analyzed with respect to splicing of minigene-derived transcripts using reverse transcription polymerase chain reaction (RT-PCR). Analysis of the c.1496-6C>A variant required modification of the pSPL3 vector, as this vector is only suitable for the splice analysis of internal exons, whereas the c.1496-6C>A variant is located at the acceptor splice site of exon 15, which is the last exon in transcript NM_003322.6. Accordingly, the second vector-resident exon along with its polyA-signal was removed from pSPL3 and replaced by a genomic segment comprising *TULP1* exon 14, intron 14, and exon 15 with its entire 3´UTR. cDNA synthesis and RT-PCR were performed following the 3´RACE protocol from Scotto-Lavino and colleagues [68].

Primers for PCR amplification, site-directed mutagenesis, cDNA synthesis, and RT-PCR are given in Supplementary Table S2.

### 4.4. Molecular Modeling of Human TULP1 and Structural Analysis of Pathological Variants

The three-dimensional structure of the C-terminal (residues 291–536) of apo human TULP1 was modeled using the experimentally solved X-ray structure with Protein Data Bank (PDB) identifier 2FIM (resolution 1.9 Å, manuscript to be published) as a template. The structure of the inositol 1,4,5-triphosphate (IP_3_)-bound form of the C-terminal domain of TULP1 was instead modeled over the template of the crystallographic structure deposited with PDB identifier 3C5N (resolution 1.8 Å, manuscript to be published). Structure refinement, in silico mutagenesis, and calculation of the Gibbs free energy variation were performed with the BioLuminate interface of Maestro (Schroedinger, New York City, NY, USA) modeling suite. As both PDB files contained the coordinates for two TULP1 molecules within the same asymmetric unit, both structures could be considered equally reliable; thus, both monomers underwent the same structure preparation and analysis pipeline, and their results were presented as average ± std. dev.

All structures were prepared using BioLuminate’s Protein Preparation protocol, briefly consisting of assigning bond orders according to the Chemical Components Dictionary database (wwPDB foundation, Piscataway, NJ, USA), and the addition of H-atoms and modeling of missing structural elements using Prime module. Specifically, loops 314–320, 359–364, and 410–418 were modeled in monomer A of apo TULP1, while loops 316–320 and 357–365 were modeled in monomer B. Loops 314–319, 359–364, and 413–416 were modeled in monomer A of the IP_3_-bound TULP1, and the loops encompassing residues 314–320, 358–364, and 468–476 were modeled in monomer B. Additionally, only the IP_3_ molecule that was present in both monomers was considered a reliable binding partner to build the IP_3_-bound state and was included in computations. When possible, the original conformation of the backbone was preserved during loop refinement, and the most common rotamer was chosen to model missing sidechains. After reconstruction of the complete polypeptide chains, the structures were refined by sampling the orientation of all water molecules present in the PDB file, prediction of the charge of residues and heteroatoms at pH 7.5 by PROPKA [69] and Epik modules, respectively, followed by assignment and optimization of H-bonds. Finally, structures underwent energy minimization based on the Optimized Potentials for Liquid Simulations 4 (OPLS4) forcefield [70] with 0.3 Å root-mean-square deviation of heavy atoms as the threshold.

All disease-associated variants were generated by in silico mutagenesis using the Residue scanning tool provided by BioLuminate, upon selection of the most common rotamer, and energy-minimized using the same forcefield and threshold as above.

The effects of amino acid substitutions (compared to the wild-type) on the Gibbs free energy of folding (∆∆G_f_^app^ = ∆G_f_^app^_mut_ − ∆G_f_^app^_WT_) and binding to IP_3_ (∆∆G_b_^app^ = ∆G_b_^app^_mut_ − ∆G_b_^app^_WT_) were computed following the thermodynamic cycle for each variant, using the Molecular Mechanics/Generalized Born and Surface Area Continuum solvation (MM/GBSA) [71] method. Such a method, though, does not account for the explicit contribution derived from conformational changes; thus, the reported free energy variations represent apparent values rather than precise thermodynamic measurables.

## 5. Conclusions

In summary, we report the largest cohort of patients with *TULP1*-associated IRD to date. Most of the patients exhibited rod-driven disease, yet a fraction of the patients exhibited cone-driven disease. Our structural analyses strongly suggest that missense variants in the Tubby domain of the TULP1 protein display different degrees of structural destabilization. Overall, our data support the hypothesis that *TULP1* variants do not fold properly and thus trigger unfolded protein response, which in turn causes ER stress and, consequently, leads to photoreceptor death.

## Figures and Tables

**Table 1:** Clinical data and genotypes of 17 patients with *TULP1*-associated inherited retinal dystrophy.

ID	Clinical Diagnosis	Age at Last Exam	Sex	BCVA [logMAR]	VF Radius [Target III4e]	ERG	*TULP1* Genotype
P1	LCA	6	f	OD 1.15 OS 0.90	OD n.d. OS ~40°	flat	c.568G>T;p.(E190*) hom
P2	LCA	12	f	OD 0.70 OS 0.50	OD ~10° OS n.d.	flat	c.901C>T;p.(Q301*) hom
P3	LCA	62	f	OD 2.30 OS 2.30	OD n.d. OS n.d.	flat	c.1523G>A;p.(R508H) hom
P4	eoRP	6	m	OD 0.40 OS 0.40	OD n.d. OS n.d.	n.d.	c.1163C>A;p.(P388Q) het c.1445G>A;p.(R482Q) het
P5	eoRP	14	m	OD 0.50 OS 0.40	OD n.d. OS ~40°	flat	c.629C>G;p.(S210*) hom
P6	eoRP	21	m	OD 0.50 OS 0.50	OD <10° OS <10°	n.d.	c.1081C>T;p.(R361*) het c.1258C>A;p.(R420S) het
P7	eoRP	25	f	OD 0.40 OS 0.60	OD <10° OS <10°	n.d.	c.1081C>T;p.(R361*) het c.1258C>A;p.(R420S) het
P8	eoRP	33	m	OD 1.30 OS 1.20	OD ~10° OS ~10°	n.d.	c.1025G>A;p.(R342Q) het c.1496-6C>A;p.(P499Lfs*143) het
P9	eoRP	34	m	OD 2.30 OS 2.30	OD n.d. OS n.d.	flat	c.1495+1G>A;p.(A442Pfs*18) hom
P10	eoRP	37	m	OD 0.60 OS 0.70	OD <10° OS <10°	flat	c.1047T>G;p.(N349K) hom
P11	eoRP	37	m	OD 1.50 OS 0.60	OD <10° OS < 10°	flat	c.1047T>G;p.(N349K) hom
P12	eoRP	38	f	OD 0.80 OS 0.70	OD n.d. OS n.d.	flat	c.1507_1521del; p.(V503_G507del) hom
P13	RP	18	f	OD 0.10 OS 0.10	OD n.d. OS ~10°	flat	c.1024C>T;p.(R342*) het c.1496-6C>A; p.(P499Lfs*143) het
P14	RP	80	m	OD 2.70 OS 2.30	OD n.d. OS n.d.	flat	c.1496-6C>A;p.(P499Lfs*143) hom
P15	CD	40	m	OD 0.40 OS 0.40	OD normal external boundaries OS normal external boundaries	dark-adapted within normal limits, light-adapted reduced	c.1201C>T;p.(Q401*) het c.797G>T;p.(G266V) het
P16	CRD	31	m	OD 1.50 OS 1.30	OD central scotoma OS central scotoma	dark-adapted slightly reduced, light-adapted reduced	c.1163C>A;p.(P388Q) hom
P17	CRD	37	f	OD 1.50 OS 2.30	OD pericentral scotoma OS pericentral scotoma	flat	c.1471T>C; p.(F491L) het c.1496-6C>A; p.(P499Lfs*143) het

f, female; m, male; BCVA, best-corrected visual acuity; ERG, electroretinography; OD, right eye; OS, left eye; VF, visual field; n.d., no data; LCA; Leber congenital amaurosis; eoRP, early-onset RP; RP, retinitis pigmentosa; CD, cone dystrophy; CRD, cone-rod dystrophy; hom, homozygous; het, heterozygous. Nucleotide and amino acid positions refer to GenBank accession numbers NM_003322.6 and NP_003313.3. Note that patients P6 and P7 are siblings, as are P10 and P11.

**Table 2:** Observed *TULP1* variants in this cohort.

cDNA Position (NM_003322.6)	Amino Acid Position (NP_003313.3)	Variant Class	HGMD Accession Number	gnomAD MAF	Observed Number of Alleles in Cohort
c.568G>T	p.(E190*)	nonsense	-	-	2
c.629C>G	p.(S210*)	nonsense	CM140491	-	2
c.797G>T	p.(G266V)	missense	CM2035471	0.0007141	1
c.901C>T	p.(Q301*)	nonsense	CM098172	-	2
c.1024C>T	p.(R342*)	nonsense	-	-	1
c.1025G>A	p.(R342Q)	missense	CM119412	0.00001988	1
c.1047T>G	p.(N349K)	missense	CM123537	0.000007953	4
c.1081C>T	p.(R361*)	nonsense	CM140477	0.000003977	2
c.1163C>A	p.(P388Q)	missense	-	-	3
c.1201C>T	p.(Q401*)	nonsense	CM2034386	-	1
c.1258C>A	p.(R420S)	missense	CM135101	0.000008988	2
c.1445G>A	p.(R482Q)	missense	CM123565	0.000003977	1
c.1471T>C	p.(F491L)	missense	CM981971	-	1
c.1495+1G>A	p.(A442Pfs*18)	splice site	CS982391	0.00001195	2
c.1496-6C>A/p.?	p.(P499Lfs*143)	splice site	CS984713	0.00008210	5
c.1507_1521del	p.(V503_G507del)	In-frame deletion	-	-	2
c.1523G>A	p.(R508H)	missense	CM2036799	0.00001066	2

HGMD, Human Gene Mutation Database [17]; gnomAD, Genome Aggregation Database [37]; MAF, minor allele frequency.

**Table 3:** Effects of *TULP1* missense variants found in this study on the apparent relative stability (∆∆G_f_^app^) and affinity for IP_3_ (∆∆G_b_^app^).

Variant [Reference]	Apo	IP_3_-Bound	
	∆∆G_f_^app^ (kcal/mol)	∆∆G_f_^app^ (kcal/mol)	IP_3_ ∆∆G_b_^app^ (kcal/mol)
**R342Q** **[46]**	6.97 ± 3.45	4.49 ± 1.53	1.10 ± 0.16
**N349K [47]**	11.55 ± 0.04	40.10 ± 26.59	−1.24 ± 0.26
**P388Q**	9.53 ± 0.30	17.97 ± 3.30	2.67 ± 3.77
**R420S [11]**	15.58 ± 5.81	14.79 ± 3.46	2.96 ± 2.60
**R482Q [48]**	24.32 ± 0.04	29.43 ± 1.70	2.69 ± 1.99
**F491L [5]**	5.44 ± 0.88	3.58 ± 1.05	0.11 ± 0.03
**R508H**	29.32 ± 9.02	29.26 ± 8.29	13.54 ± 3.30

## Data Availability

The data presented in this study are contained within the article and supplementary materials.

## Supplementary Materials

The following supporting information can be downloaded at https://www.mdpi.com/article/10.3390/ijms24032709/s1.
