# Supplementary material for: Biallelic Variants in TULP1 Are Associated with Heterogeneous Phenotypes of Retinal Dystrophy
Source: Int J Mol Sci. 2023 Jan 31;24(3):2709. doi: 10.3390/ijms24032709 (PMC9916573; doi:10.3390/ijms24032709)
Supplement: Supplementary file 1 [file ijms-24-02709-s001.zip › Supplementary Figure S1.pdf]

A

Reference protein (NP_003313.3)

1 MPLRDETLRE VWASDSGHEE ESLSPEAPRR PKQRPAPAQR LRKKRTEAPE SPCPTGSKPR
61 KPGAGRTGRP REEPSPDPAQ ARAPQTVYAR FLRDPEAKKR DPRETFLVAR APDAEDEEEE
121 EEEDEEDEEE EAEEKKEKIL LPPKKPLREK SSADLKERRA KAQGPRGDLG SPDPPPKPLR
181 VRNKEAPAGE GTKMRKTKKK GSGEADKDPS GSPASARKSP AAMFLVGEGS PDKKALKKKG
241 TPKGARKEEE EEEEAATVIK KSNQKGKAKG KGKKKAKEER APSPPVEVDE PREFVLRPAP
301 QGRTVRCRLT RDKKGMDRGM YPSYFLHLDT EKKVFLLAGR KRKRSKTANY LISIDPTNLS
361 RGGENFIGKL RSNLLGNRFT VFDNGQNPQR GYSTNVASLR QELAAVIYET NVLGFRGPRR
421 MTVIIPGMSA ENERVPIRPR N**ASDGLLVRW QNKTLESLIE LHNKPPVWND DSGSYTLNFQ**
481 **GRVTQASVKN FQIVHADDPD YIVLQFGRVA EDAFTLDYRY PLCALQAFAI ALSSFDGKLA**
541 **CE***

Protein deduced from c.1495+1G>A minigene assay

1 MPLRDETLRE VWASDSGHEE ESLSPEAPRR PKQRPAPAQR LRKKRTEAPE SPCPTGSKPR
61 KPGAGRTGRP REEPSPDPAQ ARAPQTVYAR FLRDPEAKKR DPRETFLVAR APDAEDEEEE
121 EEEDEEDEEE EAEEKKEKIL LPPKKPLREK SSADLKERRA KAQGPRGDLG SPDPPPKPLR
181 VRNKEAPAGE GTKMRKTKKK GSGEADKDPS GSPASARKSP AAMFLVGEGS PDKKALKKKG
241 TPKGARKEEE EEEEAATVIK KSNQKGKAKG KGKKKAKEER APSPPVEVDE PREFVLRPAP
301 QGRTVRCRLT RDKKGMDRGM YPSYFLHLDT EKKVFLLAGR KRKRSKTANY LISIDPTNLS
361 RGGENFIGKL RSNLLGNRFT VFDNGQNPQR GYSTNVASLR QELAAVIYET NVLGFRGPRR
421 MTVIIPGMSA ENERVPIRPR N**PTISCCSSA AWRRTPSP***

B

Reference protein (NP_003313.3)

1 MPLRDETLRE VWASDSGHEE ESLSPEAPRR PKQRPAPAQR LRKKRTEAPE SPCPTGSKPR
61 KPGAGRTGRP REEPSPDPAQ ARAPQTVYAR FLRDPEAKKR DPRETFLVAR APDAEDEEEE
121 EEEDEEDEEE EAEEKKEKIL LPPKKPLREK SSADLKERRA KAQGPRGDLG SPDPPPKPLR
181 VRNKEAPAGE GTKMRKTKKK GSGEADKDPS GSPASARKSP AAMFLVGEGS PDKKALKKKG
241 TPKGARKEEE EEEEAATVIK KSNQKGKAKG KGKKKAKEER APSPPVEVDE PREFVLRPAP
301 QGRTVRCRLT RDKKGMDRGM YPSYFLHLDT EKKVFLLAGR KRKRSKTANY LISIDPTNLS
361 RGGENFIGKL RSNLLGNRFT VFDNGQNPQR GYSTNVASLR QELAAVIYET NVLGFRGPRR
421 MTVIIPGMSA ENERVPIRPR NASDGLLVRW QNKTLESLIE LHNKPPVWND DSGSYTLNFQ
481 GRVTQASVKN FQIVHADD**PD YIVLQFGRVA EDAFTLDYRY PLCALQAFAI ALSSFDGKLA**
541 **CE***

Protein deduced from c.1496-6C>A minigene assay

1 MPLRDETLRE VWASDSGHEE ESLSPEAPRR PKQRPAPAQR LRKKRTEAPE SPCPTGSKPR
61 KPGAGRTGRP REEPSPDPAQ ARAPQTVYAR FLRDPEAKKR DPRETFLVAR APDAEDEEEE
121 EEEDEEDEEE EAEEKKEKIL LPPKKPLREK SSADLKERRA KAQGPRGDLG SPDPPPKPLR
181 VRNKEAPAGE GTKMRKTKKK GSGEADKDPS GSPASARKSP AAMFLVGEGS PDKKALKKKG
241 TPKGARKEEE EEEEAATVIK KSNQKGKAKG KGKKKAKEER APSPPVEVDE PREFVLRPAP
301 QGRTVRCRLT RDKKGMDRGM YPSYFLHLDT EKKVFLLAGR KRKRSKTANY LISIDPTNLS
361 RGGENFIGKL RSNLLGNRFT VFDNGQNPQR GYSTNVASLR QELAAVIYET NVLGFRGPRR
421 MTVIIPGMSA ENERVPIRPR NASDGLLVRW QNKTLESLIE LHNKPPVWND DSGSYTLNFQ
481 GRVTQASVKN FQIVHADD**LR PRGGGRLHPR LPVPAVRPAG LRHRPLQFRR EAGLRVTPAA**
541 **PQRPQSPSAW GKGFSGGWQG PSSKAPAENC SCVGADLSLP LGDLRPLPSL AQAEAGGART**
601 **AGRTEMKNIW SWSRTSGLGA RLRRCAPLLP APQSLPVREQ ***

Supplementary Figure S1: Deduced outcome of the two splice variants c.1495+1G>A (A) and c.1496-6C>A (B). The amino acids of the reference protein that are lost due to the mutation are highlighted in green. The amino acids in the mutant protein that are newly formed by the frameshift are highlighted in red.
