# Supplementary material for: Biallelic Variants in TULP1 Are Associated with Heterogeneous Phenotypes of Retinal Dystrophy
Source: Int J Mol Sci. 2023 Jan 31;24(3):2709. doi: 10.3390/ijms24032709 (PMC9916573; doi:10.3390/ijms24032709)
Supplement: Supplementary file 1 [file ijms-24-02709-s001.zip › Supplementary Figure S2.pdf]

H. sapiens	227	GEGSPDKKALKKKGTPKGARKEEEEEEE------AATVIKKSNQK**G**KAKG	270
P. troglodytes	227	GEGSPDKKALKKKGTPKGARKEEEEEEE------AATVTKNSNQK**G**KAKG	270
M. mulatta	223	GEGSPDKKALKKKGTPKGGRKEEEEEEE------AATVTKNSNQK**G**KAKG	266
C. lupus	229	GEEGPAEKAQKKKGISKGSEEQKKEEEEE-GGEVAGVVTKNSNQK**G**KAKG	277
B. taurus	226	PGECPAEKTLKKKGTPKGSEEEKKEEGEE-DDEAAAAMTKNSNQK**G**KAKG	274
M. musculus	222	GEGGAAEKGVKKKGPPKGSEEEKKEEEEEVEEEVASAVMKNSNQK**G**RAKG	271
R. norvegicus	172	GEGGTAEKGVKKK------------------------VMRNSNQK**G**RAKG	197
G. gallus	51	GGDPPRERKTRKKAPKTAESEEETLETQ----------QKNSNKK**G**K-GK	89
X. tropicalis	208	KGDVSKDVKGKKKGLAKSNEEEEEEEEL----------TKNTNKK**G**KRGK	247

Supplementary Figure S2: Sequence conservation of the G266 residue in orthologous TULP1 protein sequences. The conserved glycine residue is shown in red. Orthologous sequences were obtained from NCBI. NCBI reference sequences are as follows: *Homo sapiens* (NP_003313.3), *Pan troglodytes* (XP_001172351.1), *Macaca mulatta* (XP_001111988.2), *Canis lupus* (XP_538879.3), *Bos taurus* (NP_001193657.1), *Mus musculus* (NP_067453.1), *Rattus norvegicus* (NP_001101112.1), *Gallus gallus* (NP_989946.1), *Xenopus tropicalis* (NP_001016482.1).
