# Supplementary material for: Biallelic Variants in TULP1 Are Associated with Heterogeneous Phenotypes of Retinal Dystrophy
Source: Int J Mol Sci. 2023 Jan 31;24(3):2709. doi: 10.3390/ijms24032709 (PMC9916573; doi:10.3390/ijms24032709)
Supplement: Supplementary file 1 [file ijms-24-02709-s001.zip › Supplementary Table S1.pdf]

Supplementary Table S1. Effects of known TULP1 missense variants (retrieved from HGMD and literature) on the apparent relative stability ($\Delta\Delta G_f^{app}$) and affinity for IP3 ($\Delta\Delta G_b^{app}$).

Variant	HGMD accession number	Apo	IP3-bound	
		$\Delta\Delta G_f^{app}$ (kcal/mol)	$\Delta\Delta G_f^{app}$ (kcal/mol)	IP3 $\Delta\Delta G_b^{app}$ (kcal/mol)
**R311Q**	CM119411	18.95 ± 6.75	31.86 ± 3.60	14.79 ± 3.50
**R311W**	CM161804	23.24 ± 12.30	72.70 ± 5.86	21.34 ± 11.72
**G319D**	CM155321	-0.22 ± 1.41	9.17 ± 1.37	1.51 ± 0.58
**Y321D**	CM1310211	0.41 ± 7.08	1.33 ± 7.33	1.86 ± 0.33
**R342Q**	CM119412	6.97 ± 3.45	4.49 ± 1.53	1.10 ± 0.16
**N349K**	CM123537	11.55 ± 0.04	40.10 ± 26.59	-1.24 ± 0.26
**D355V**	CM1310212	7.01 ± 1.39	13.00 ± 2.59	-0.71 ± 0.21
**G363R**	CM156228	-3.84 ± 2.83	2.31 ± 3.36	-0.72 ± 0.07
**G368W**	CM040810	2690.85 ± 2133.29	2757.74 ± 2.93	0.26 ± 0.38
**T380A**	CM100226	13.03 ± 5.77	11.28 ± 3.92	1.47 ± 2.06
**F382S**	CM045204	24.51 ± 0.12	21.52 ± 3.85	0.06 ± 0.11
**G385R**	CM155827	18.20 ± 9.36	47.97 ± 0.41	5.60 ± 8.89
**P388S**	-*	9.76 ± 1.11	9.70 ± 2.07	0.56 ± 0.77
**R400Q**	CM095222	22.01 ± 7.54	20.05 ± 5.49	7.70 ± 9.69
**R400W**	CM040811	44.39 ± 19.59	45.65 ± 0.23	12.05 ± 15.83
**A405P**	CM1514188	57.75 ± 7.41	67.28 ± 6.22	0.10 ± 0.11
**R416C**	CM149203	10.78 ± 4.47	15.51 ± 6.02	4.98 ± 5.47
**R419Q**	CM168239	7.92 ± 1.03	14.25 ± 0.74	2.74 ± 0.35
**R419W**	CM1412147	7.11 ± 3.20	24.70 ± 8.88	3.64 ± 2.11
**R420S**	CM135101	15.60 ± 5.83	14.76 ± 3.44	2.96 ± 2.60
**R420P**	CM981969	72.36 ± 8.07	50.89 ± 53.49	2.87 ± 2.42
**P426L**	CM1310210	93.49 ± 56.59	53.71 ± 15.87	5.03 ± 7.09
**R438P**	CM170105	37.54 ± 6.17	31.87 ± 1.63	0.21 ± 0.67
**I459K**	CM981970	21.69 ± 0.95	25.21 ± 4.96	-2.14 ± 1.92
**I459T**	CM141764	11.28 ± 0.24	12.50 ± 0.09	0.20 ± 0.30
**L461V**	CM076576	20.45 ± 1.97	22.71 ± 0.20	0.25 ± 0.34
**R482W**	CM073391	34.13 ± 0.76	45.28 ± 3.31	5.48 ± 5.93
**R482Q**	CM123565	24.32 ± 0.04	29.43 ± 1.70	2.69 ± 1.99
**K489R**	CM984710	-16.79 ± 4.35	-6.91 ± 9.92	14.24 ± 0.41
**F491L**	CM981971	5.44 ± 0.88	3.58 ± 1.05	0.11 ± 0.03
**Q492R**	CM161805	27.37 ± 7.64	23.38 ± 8.95	-1.78 ± 0.06
**A496T**	CM984711	-3.16 ± 1.79	-1.28 ± 3.16	-2.44 ± 3.43
**P499S**	CM1514189	2.82 ± 0.38	2.36 ± 0.55	-1.10 ± 1.53
**F506L**	CM1310209	6.14 ± 0.77	8.80 ± 0.30	-0.01 ± 0.06
**P521S**	CM168614	17.55 ± 2.43	17.88 ± 0.06	0.45 ± 0.61
**C523Y**	CM229184	149.08 ± 23.84	121.35 ± 11.95	-0.49 ± 0.69
**I530M**	CM1618292	-3.00 ± 0.76	-0.04 ± 0.08	-1.06 ± 0.58
**F535S**	CM1312806	18.91 ± 5.52	19.36 ± 2.16	0.66 ± 0.83

HGMD, Human Gene Mutation Database [17].*Variant has no HGMD accession number but is described in [56].
