# Supplementary material for: Biallelic Variants in TULP1 Are Associated with Heterogeneous Phenotypes of Retinal Dystrophy
Source: Int J Mol Sci. 2023 Jan 31;24(3):2709. doi: 10.3390/ijms24032709 (PMC9916573; doi:10.3390/ijms24032709)
Supplement: Supplementary file 1 [file ijms-24-02709-s001.zip › Supplementary Table S2.pdf]

Supplementary Table S2. Primers used in this study.

**Primer name**	**Sequence (5´→3´)**	**Purpose**
TULP1-ex14-*Not*I	**TATATATA***GCGGCCGC*AAGCCCCATTCTCACAGATG	Primer pair to amplify *TULP1* exon 14 with adjacent intronic sequence
TULP1-ex14-*Bam*HI	**TATATATA***GGATCC*GGGCCAGTGTAACTTCCTGA	
TULP1-ex14-*Not*I	**TATATATA***GCGGCCGC*AAGCCCCATTCTCACAGATG	Primer pair to amplify *TULP1* exon 14, intron 14, exon 15 and the 3´UTR
TULP1-3´UTR-*Bgl*II	**TATATATA***AGATCT*GGGGTTGAGACTTCCAGCTC	
TULP1-IVM-c.1495+1GtoA-f	GTCCACGCTGATGACCATGAGTACCTGAGGGC	IVM primer pair to introduce the c.1495+1G>A variant
TULP1-IVM-c.1495+1GtoA-r	GCCCTCAGGTACTCATGGTCATCAGCGTGGAC	
TULP1-IVM-c.1496CtoA-f	CCACCCCGTCTTGTGTACATAGCCGACTATATCGTG	IVM primer pair to introduce the c.1496-6C>A variant
TULP1-IVM-c.1496CtoA-r	CACGATATAGTCGGCTATGTACACAAGACGGGGTGG	
pSPL3-SA2	ATCTCAGTGGTATTTGTGAGC	cDNA synthesis for exon 14 minigene construct
Oligo dT	TTTTTTTTTTTTTTTTTT	3´RACE for exon14-exon 15 minigene construct
pSPL3-SD6	TCTGAGTCACCTGGACAACC	Primer pair for 1st 3´RACE RT-PCR
TULP1-3´RACE-nested-R1	TTGGCCAAGGACGCGGCTTTATTG	
pSPL3-cDNA-f	TGGACAACCTCAAAGGCACC	Primer pair for 2nd 3´RACE RT-PCR
TULP1-3´RACE-nested-R2	GGTAGTCTAGGGTGAAGGCGTC	

Binding sites for restriction enzymes are indicated in italics. 5´-extension tails to the restriction sites are indicated in bold.
