# Supplementary material for: Biallelic Variants in TULP1 Are Associated with Heterogeneous Phenotypes of Retinal Dystrophy
Source: Int J Mol Sci. 2023 Jan 31;24(3):2709. doi: 10.3390/ijms24032709 (PMC9916573; doi:10.3390/ijms24032709)
Supplement: Supplementary file 1 [file ijms-24-02709-s001.zip › Supplementary videos-Legends.pdf]

Supplementary Video S1: Three-dimensional structure of the Tubby domain of IP_3-bound TULP1. Protein structure is displayed as cartoons in a blue-to-red color scheme according to the sequence, secondary structure elements are labelled, IP_3 is framed and represented as sticks with C atoms in black, P atoms in violet and O atoms in red.

Supplementary Video S2: Structural localization of the novel TULP1 missense variants identified in this study. Protein structure is displayed as grey cartoons, novel TULP1 missense variants are represented as sticks with C atoms in cyan, N atoms in blue and O atoms in red, IP_3 is shown as sticks with C atoms in black, P atoms in violet and O atoms in red.

Supplementary Video S3: Structural localization of previously known TULP1 missense variants. Protein structure is displayed as grey cartoons, previously known TULP1 missense variants are represented as yellow sticks, IP_3 is shown as sticks with C atoms in black, P atoms in violet and O atoms in red.
